# Solid Polymeric Nanoparticles of Albendazole: Synthesis, Physico-Chemical Characterization and Biological Activity

**DOI:** 10.3390/molecules25215130

**Published:** 2020-11-04

**Authors:** Roxana Racoviceanu, Cristina Trandafirescu, Mirela Voicu, Roxana Ghiulai, Florin Borcan, Cristina Dehelean, Claudia Watz, Zoltán Aigner, Rita Ambrus, Dorina Elena Coricovac, Denisa Cîrcioban, Alexandra Mioc, Camelia Alexandrina Szuhanek, Codruţa Şoica

**Affiliations:** 1 Department of Pharmaceutical Chemistry, Victor Babeș University of Medicine and Pharmacy, 2nd Eftimie Murgu Sq., 300041 Timisoara, Romania; babuta.roxana@umft.ro (R.R.); trandafirescu.cristina@umft.ro (C.T.); codrutasoica@umft.ro (C.Ş.); 2 Department of Pharmacology and Clinical Pharmacy, Victor Babeș University of Medicine and Pharmacy, 2nd Eftimie Murgu Sq., 300041 Timisoara, Romania; 3 Department of Analytical Chemistry, Victor Babeș University of Medicine and Pharmacy, 2nd Eftimie Murgu Sq., 300041 Timisoara, Romania; fborcan@umft.ro (F.B.); circioban.denisa@umft.ro (D.C.); 4 Department of Toxicology, Victor Babeș University of Medicine and Pharmacy, 2nd Eftimie Murgu Sq., 300041 Timisoara, Romania; cadehelean@umft.ro (C.D.); dorinacoricovac@umft.ro (D.E.C.); 5 Department of Pharmaceutical Physics, Victor Babeș University of Medicine and Pharmacy, 2nd Eftimie Murgu Sq., 300041 Timisoara, Romania; farcas.claudia@umft.ro; 6 Institute of Pharmaceutical Technology and Regulatory Affairs, Faculty of Pharmacy, University of Szeged, 6th Eotvos Str., 6720 Szeged, Hungary; aigner@pharm.u-szeged.hu (Z.A.); arita@pharm.u-szeged.hu (R.A.); 7 Department of Anatomy, Physiology and Physiopathology, Victor Babeș University of Medicine and Pharmacy, 2nd Eftimie Murgu Sq., 300041 Timisoara, Romania; alexandra.petrus@umft.ro; 8 Department of Orthodontics, Victor Babeș University of Medicine and Pharmacy, 9th Revolutiei din 1989 Bvd, 300041 Timisoara, Romania; cameliaszuhanek@umft.ro

**Keywords:** albendazole, polyurethane, nanoparticles, cell viability

## Abstract

Albendazole is a benzimidazole derivative with documented antitumor activity and low toxicity to healthy cells. The major disadvantage in terms of clinical use is its low aqueous solubility which limits its bioavailability. Albendazole was incorporated into stable and homogeneous polyurethane structures with the aim of obtaining an improved drug delivery system model. Spectral and thermal analysis was used to investigate the encapsulation process and confirmed the presence of albendazole inside the nanoparticles. The in vitro anticancer properties of albendazole encapsulated in polyurethane structures versus the un-encapsulated compound were tested on two breast cancer cell lines, MCF-7 and MDA-MB-231, in terms of cellular viability and apoptosis induction. The study showed that the encapsulation process enhanced the antitumor activity of albendazole on the MCF-7 and MDA-MB-23 breast cancer lines. The cytotoxic activity manifested in a concentration-dependent manner and was accompanied by changes in cell morphology and nuclear fragmentation.

## 1. Introduction

Albendazole (ABZ), methyl[5-(propylthio)-1-*H*-benzimidazol-2-yl]carbamate, is one of the most effective broad-spectrum anti-helminthic agents used in human and veterinary chemotherapy of various intestinal and systemic parasitosis [1]. The drug has a very low intestinal absorption caused by poor aqueous solubility and suffers an extensive first-pass metabolism occurring in enterocytes and liver cells; both these unfavorable properties result in low and erratic bioavailability, limiting the use of ABZ as an antiparasitic agent [2,3]. The ability of ABZ to inhibit the tubulin chains of parasites has been exploited in recent years in the investigation of its anticancer potential. ABZ disrupts the mitosis process, inhibiting microtubule formation and interfering with microtubule function [4]. The ABZ antitumor properties have been reported by several researches who revealed an anticancer activity against adrenocortical carcinoma, lung, ovarian and brain cancer and melanoma [5,6,7]. Furthermore, the potential of ABZ to sensitize cancer cells to ionizing radiation was shown in a study conducted by Patel et al., suggesting the potential use of ABZ in combination with radiation therapy for the treatment of brain metastases [8]. Ehteda et al. revealed the synergistic effect of ABZ and 2-methoxyestradiol against HCT-116 tumor bearing nude mice [9]; researches also highlighted the property of ABZ to induce oxidative stress thus promoting DNA fragmentation, triggering apoptosis and inducing cell death [10].

Despite its promising potential as an anticancer drug with a favorable toxicological profile, the clinical use of ABZ raises some challenges due to its poor gastrointestinal absorption and bioavailability [8].

One approach to overcome shortcomings of drugs and their conventional pharmaceutical formulations is provided by nanotechnology. Numerous reports in the field of biomedical nanoparticles (NP) have been documented for their usefulness in improving bioavailability, and subsequently, in the treatment and prevention of many diseases [11,12,13]; moreover, encapsulation of anticancer drugs in nanocarriers may provide efficient targeted delivery of the chemotherapeutic agent into the cancer cells [14].

In recent years, the literature reports several studies focused on the development of novel nanoformulations containing ABZ, such as albumin nanoparticles [4], solid-lipid nanoparticles [15], chitosan-tripolyphosphate nanoparticles [16] and chitosan-PLGA nanoparticles [17] which were designed and tested as anticancer agents with promising results on various tumor cell lines.

Polymeric delivery systems based on synthetic polymers are currently under investigation due to their wide range of biomedical applications. Synthetic polymers are preferred to natural ones, due to several advantages such as controlled synthesis manner, well-established composition, molecular weight, predictable solubility and degradability; the interest of using polyurethanes (PU) in the biomedical field is justified by their useful properties, in particular their biocompatibility [12,18].

In the current work we set our goal to develop a novel delivery system for ABZ in order to enable and enhance its antitumor activity. Albendazole-loaded polyurethane (ABZ-PU) nanoparticles were prepared using interfacial polycondensation combined with spontaneous emulsification technique and their solid state physico-chemical properties were characterized by thermal and spectroscopic techniques. Subsequently, the anti-proliferative effect of ABZ alone and embedded in PU nanoformulations was assessed by in vitro analysis.

## 2. Results

### 2.1. Encapsulation Efficiency

The UV-Vis evaluation indicates a high binding affinity (62.4%). The mathematical interpretation of this entrapment percentage allowed us to calculate the concrete amount of ABZwhich was embedded in each mg of the PU matrix. The ABZ amount relative to the mass of the dried sample was calculated as 0.2 mg ABZ per mg nanoparticles.

### 2.2. Evaluation of Particle Size and Polydispersity Index

The evaluation of particle size and polydispersity index indicated that empty PU particles have a diameter of 112.3 ± 11.7 nm and a polydispersity index of 0.2. The ABZ-PU particles were characterized by a diameter of 128.1 ± 12.2 nm and a polydispersity index of 0.3. The larger diameter of ABZ-PU particles could be attributed to the incorporation of ABZ into the PU particles; the diameters recorded for the synthesized particles fit them at the borderline between the micro (100 nm –100 µm) and the nano (1–100 nm) domains. Being much closer to the nano domain than to the micro one, we will treat them in the following as nanostructures.

### 2.3. Evaluation of Zeta Potential

We have obtained PU empty particles with a positive surface charge of 32.13 ± 1.61 mV, and ABZ-PU particles with a positive surface charge of 35.26 ± 1.97 mV, indicating a very low tendency to conglomerate or to form clusters.

### 2.4. Thermal Analysis

#### 2.4.1. DSC/TG/DTG Analysis

**Figure 1:**
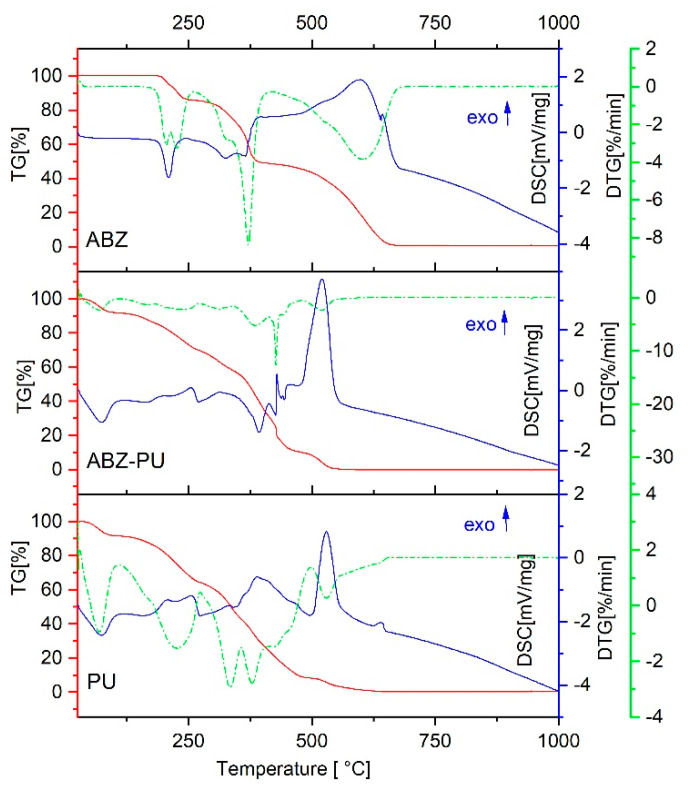
Thermal analysis—TG (red), DSC (blue) and DTG (green).

Figure 1 depicts the thermal analyses results for the pure ABZ, empty PU particles and ABZ-PU particles. The DSC thermal curve for ABZ showed an endothermic effect around 200 °C, attributed to its melting point. As one can notice on the TG/ DTG curves, shortly after the melting of ABZ, a first stage of mass loss started; the decomposition of ABZ occurs in two stages of mass loss, the second one around 300 °C. After the temperature reached 400 °C, the mass dropped to zero accompanied by a powerful exothermic effect this indicated sample combustion without residue.

For the PU nanoparticles one can notice an endothermic peak and a small mass loss around 70 °C, attributed to the water removal from the system. The melting starts above 200 °C and is quickly followed by decomposition as the TG/DTG curves suggest; in this case, the TG curve does not provide much information but the DTG curve clearly illustrates the steps of PU decomposition. Two exothermic effects can be seen above 300 °C accompanied by mass loss; basically, at 600 °C the mass was zero, suggesting the complete burning of the PU material.

The ABZ-PU thermal behavior was similar to the empty polyurethane structures, as revealed by the DSC and TG profiles; the DTG thermogram indicates a steep decomposition at around 400 °C.

#### 2.4.2. Hot-Stage Microscopy (HSM)

**Figure 2:**
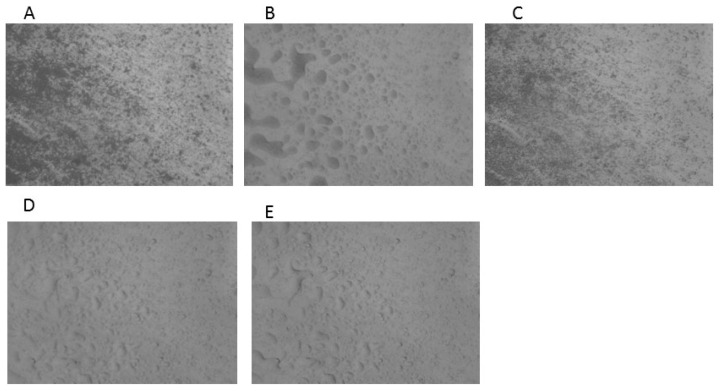
HSM micrographs for ABZ at different temperatures: (**A**)—ABZ at 25 °C; (**B**)—ABZ at 25 °C—back cooled product; (**C**)—ABZ at 195 °C—melting starts; (**D**)—ABZ at 197 °C—melted; (**E**)—ABZ at 200 °C—melted. The magnification in the photographs was 59.7×.

**Figure 3:**
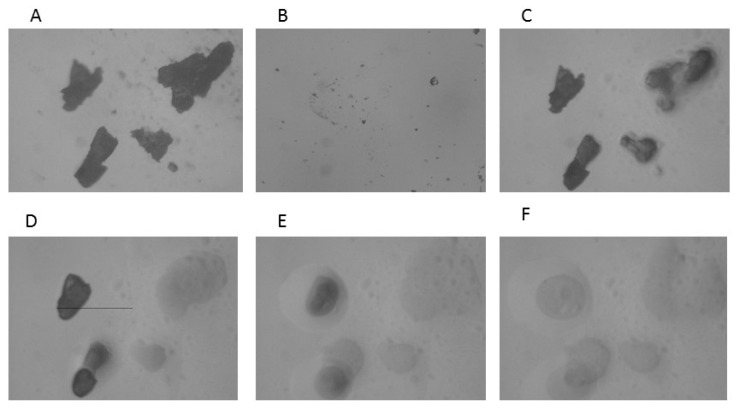
HSM micrographs for PU at different temperatures: (**A**)—PU at 25 °C; (**B**)—PU at 25 °C—back cooled product; (**C**)—PU at 245 °C—melting starts; (**D**)—PU at 250 °C—partially melted; (**E**)—PU at 255 °C—partially melted; (**F**)—PU at 260 °C—melted. The magnification in the photographs was 59.7×.

**Figure 4:**
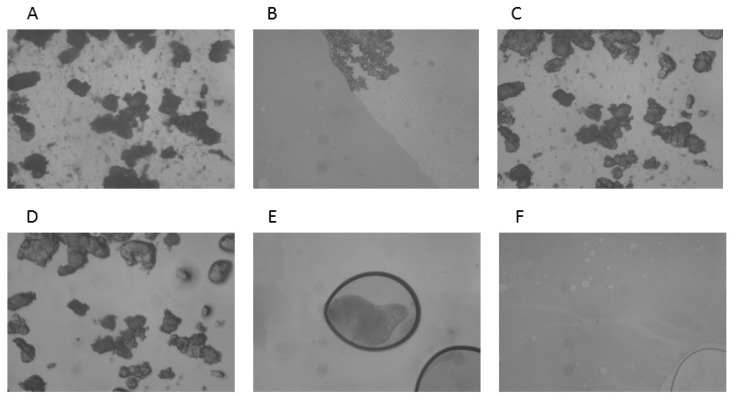
HSM micrographs for ABZ-PU particles at different temperatures: (**A**)—ABZ-PU at 25 °C; (**B**)—ABZ-PU at 25 °C—back cooled product; (**C**)—ABZ-PU at 160 °C—some particles melted; (**D**)—ABZ-PU at 230 °C—melting starts for other particles; (**E**)—ABZ-PU at 245 °C—melted; (**F**)—ABZ-PU at 250 °C—melted. The magnification in the photographs was 59.7×.

The results of the HSM analysis are depicted in Figure 2, Figure 3 and Figure 4; the examination of ABZ sample (Figure 2) revealed that the melting process started at 195 °C and all the material was in the melted state at 197–200 °C, which was in good agreement with the results obtained by the DSC analysis. HSM analysis of PU (Figure 3) indicated that the fusion process started at 245 °C; the material maintained under heating presented a partially melted aspect at 250–255 °C and the melting process was complete at 260 °C.

The HSM analysis of ABZ-PU nanoparticles (Figure 4) revealed two melting events; namely, at 160 °C some particles started to melt and at 230 °C melting was noticed for some other particles. The first melting event may be due to the existence of ABZ particles adsorbed onto the surface of NPs through hydrogen bonding while the second event was characteristic for the ABZ-PU NP.

### 2.5. Morphological Characterization

**Figure 5:**
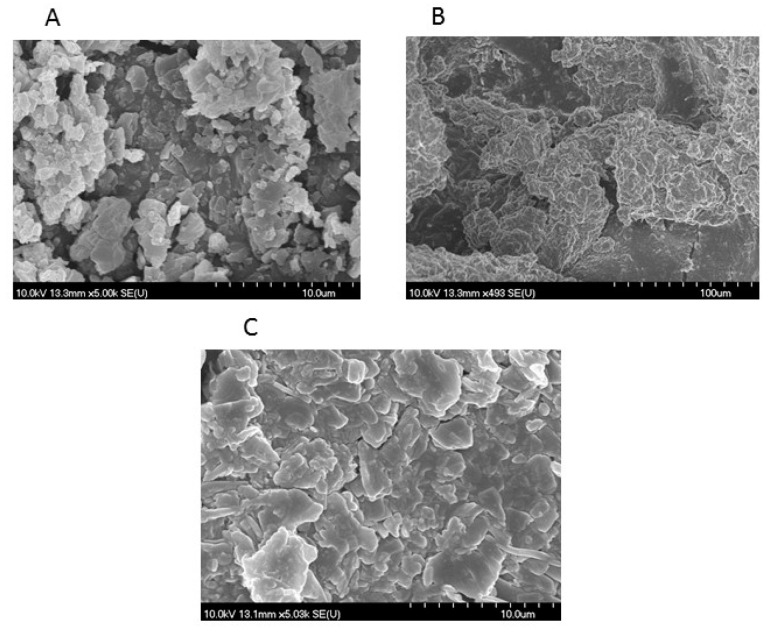
SEM microphotographs, (**A**)—ABZ; (**B**)—empty PU structures; (**C**)—ABZ-PU particles.

The morphology of the polyurethane delivery nanosystem was investigated by scanning electron microscopy (SEM). The morphological differences between the samples are depicted in Figure 5. The photomicrograph of pure ABZ (A) revealed the presence of crystalline particles with irregular shape, rough surface and a strong tendency to aggregate. The pure PU (B) appeared as a smooth material with plain surfaces. ABZ-PU nanoparticles (C) revealed significant morphological changes in their shape and overall aspect compared to the pure drug.

### 2.6. Spectroscopic Characterization

#### 2.6.1. FTIR Spectroscopy

**Figure 6:**
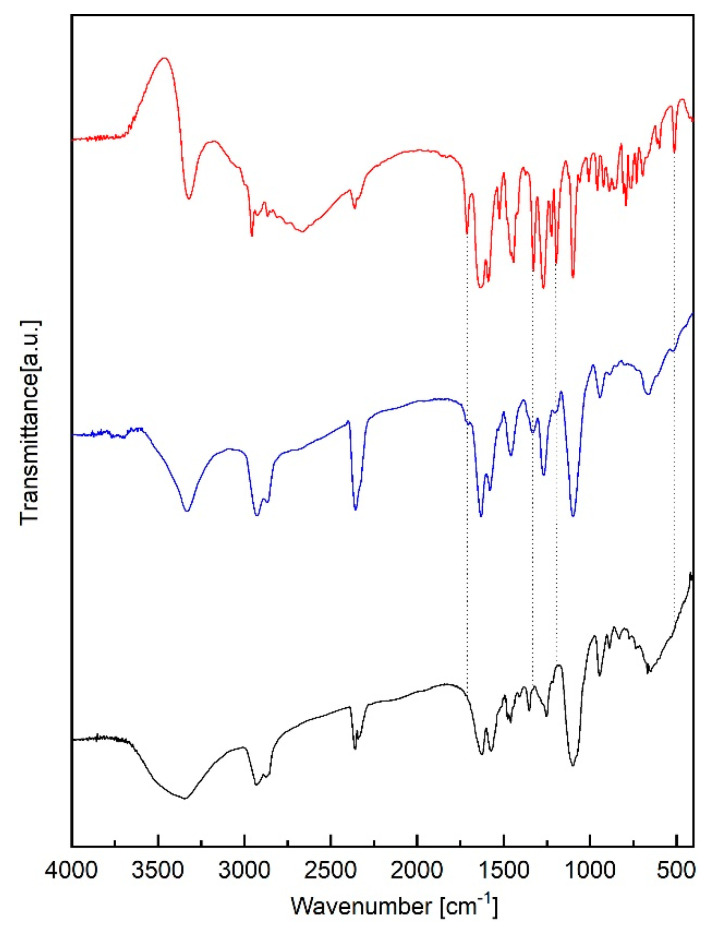
FTIR spectra of ABZ (red), ABZ-PU (blue) and PU (black).

FTIR spectra of ABZ, PU and ABZ-PU nanoparticles are shown in Figure 6. Albendazole as raw material was previously characterized by our team [19] and the obtained spectra was in good agreement with the existent literature data [2,20,21]. The main characteristic peaks observed in the ABZ spectrum were assigned as follows: 3323.35 cm^−1^, N-H stretching in carbamate; 2956.87 cm^−1^ and 2871.32 cm^−1^, C-H aliphatic stretching vibration; 2663.69 cm^−1^ –NH intramolecular in imidazole; 1712.79 cm^−1^, bending vibration of C=O bond in carbamate; 1635 cm^−1^, C=C aromatic and -N-H out of the plane bending in benzimidazole; 1523.76 cm^−1^ stretching vibration of C=N group. The bands located at 1442.75 and 1327.03, could be attributed to C-N and C-O stretching vibration. The five bands located between 1222.87 and 958.62 correspond to CH and NH in-plane bending vibrations and CH- deformation. The last region of bands, 923.90–597.93 cm^−1^ consist in skeletal vibrations, CH out of plane bending, NH_2_ rocking and C-S stretching vibrations [22,23,24,25].

The main characteristic bands observed in the pure PU spectrum were assigned as follows: the N-H characteristic band at 3500–3250 cm^−1^ with a peak at around 3346; the C-H band at 3000–2750 cm^−1^ with a peak at around 2873, and the band corresponding to the fingerprint region, at 750–1750 cm^−1^, in which C=O, C=C and C-O groups exhibited their peaks at 1624, 1572, 1097, respectively. The absence of the absorption band at 2200–2300 cm^−1^ attributable to the free isocyanate group indicated that the polymerization process was completed. One can notice the presence of a small amount of water in the system as indicated by the presence of an absorption band at 1624 cm^−1^.

The FTIR spectra of ABZ-PU NP did exhibit four attenuated characteristic absorption bands of pure ABZ (1712.79, 1327.03, 1195.87 and 597.93 cm^−1^), thus indicating an incomplete encapsulation in the core of PU NP.

#### 2.6.2. X-ray Spectroscopy

**Figure 7:**
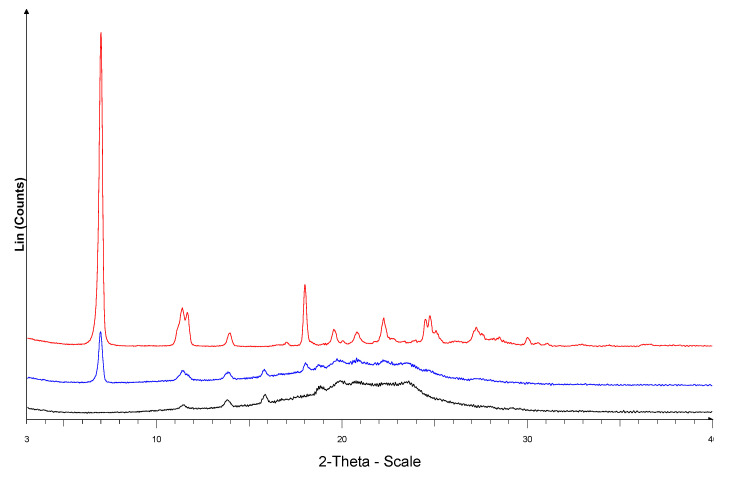
PXRD of ABZ (red), empty PU structures (black) and ABZ-PU (blue) particles.

The X-ray spectra of the ABZ, PU and ABZ-PU particles are shown in Figure 7. ABZ is a crystalline material, with characteristic peaks at 6.97; 11.41; 11.65; 13.93; 17.99; 19.58; 20.81; 22.25; 24.50; 24.74 and 27.26 2θ degrees, in accordance with the data reported in the literature [21,26,27]. PU is a semicrystalline material with some characteristic peaks at 11.41; 13.78; 15.85 and 18.83 2θ degrees and an amorphous background between 16–26 2θ degrees.

The ABZ-PU diffractogram is very similar in shape to the corresponding PU diffractogram; however, some ABZ characteristic peaks can be noticed thus indicating the presence of some non-encapsulated ABZ material.

### 2.7. In Vitro Dissolution Studies

**Figure 8:**
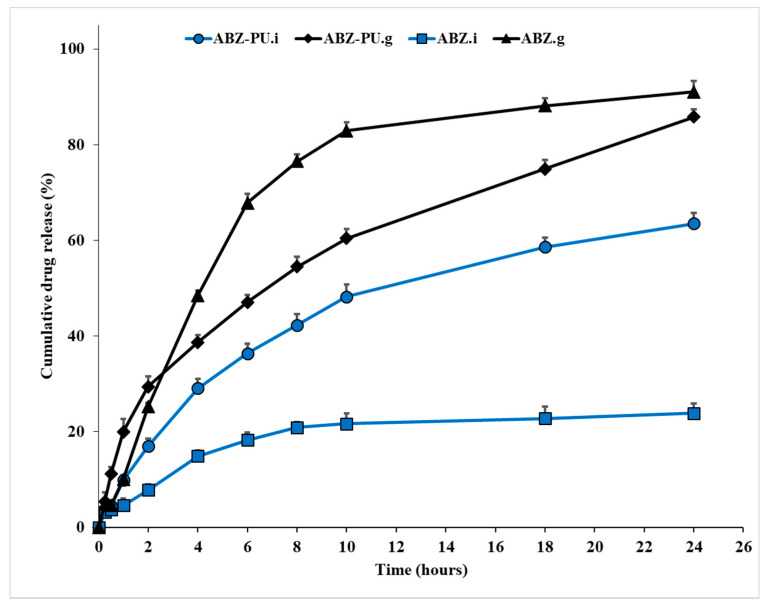
In vitro dissolution profiles of ABZ as pure drug in simulated gastric medium (ABZ.g), simulated intestinal medium (ABZ.i), PU-loaded drug in simulated gastric medium (ABZ-PU.g) and simulated intestinal medium (ABZ-PU.i).

**Figure 9:**
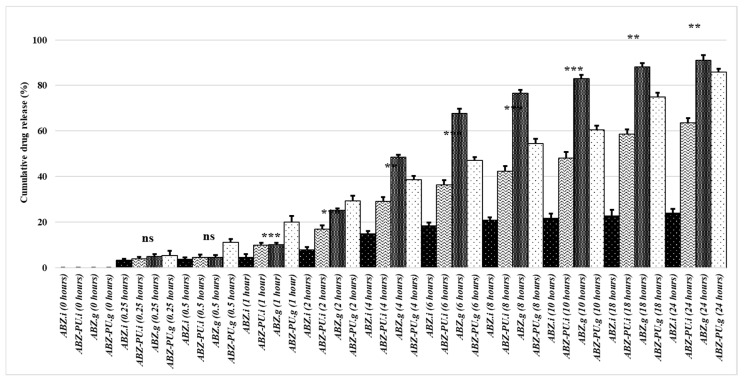
Comparative dissolution studies between pure ABZ and ABZ-PU formulations in simulated gastric/intestinal media, respectively, at different time points using one way ANOVA test. Values are mean ± SD. *p* > 0.05 not significant (ns), *p* ≤ 0.05 (*), *p* ≤ 0.01 (**), and *p* ≤ 0.001 (***).

The in vitro dissolution studies provided the release behavior of both pure and PU-loaded ABZ; the results are displayed in Figure 8 and Figure 9. The calculation of the ABZ amount which was either directly dissolved or released from the PU particles and then solubilized in the respective dissolution medium was based on the calibration lines obtained for the dissolution of ABZ in simulated gastric/intestinal medium, respectively, at the appropriate wavelengths (see Section 4).

The calibration lines correlate the amount of solubilized ABZ with the solution UV absorption and are characterized by the following equations:

Simulated gastric medium: y = 0.0434x, R^2^ = 0.992

Simulated intestinal medium: y = 0.0534x, R^2^ = 0.999

One can notice that the pure ABZ exhibits a poor dissolution profile in neutral environment (simulated intestinal medium, pH = 7) and a dramatic solubility increase in acid medium (simulated gastric medium, pH = 1.2). This behavior emphasizes the fact that ABZ aqueous solubility is significantly dependent on the pH value of the dissolution madium.

After PU encapsulation the dissolution profile of the drug improves in the neutral environment; in gastric medium however, the shape of the dissolution curve changes, revealing a lower cumulative drug release compared to the pure drug over 24 h. Nevertheless, the dissolution profile in gastric medium indicates the sustained release of the drug over the studied time interval (24 h). A similar sustained drug release can be seen in intestinal medium as well but with lower cumulative drug release compared to the gastric medium. The spectra drawn for each collected sample over the entire UV-Vis range of wavelengths revealed a single absorption maximum for each medium, respectively, 292 nm for the simulated gastric medium and 296 nm for the simulated intestinal medium, identical with the respective values recorded for pure ABZ.

### 2.8. In Vitro Anti-Proliferative Evaluation

#### 2.8.1. Cell Viability

The MTT test was performed in order to evaluate the effect induced by five different concentrations (5, 15, 25, 50, 100 µM) of test samples (ABZ, PU, ABZ-PU, respectively) on two genotypically different human breast adenocarcinoma cell lines (MCF7–ER+ and MDA-MB-231–ER−).

**Figure 10:**
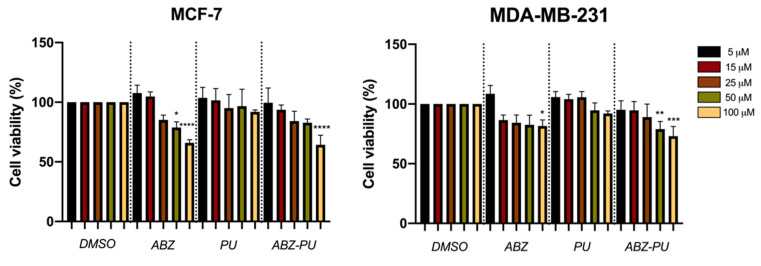
The viability percentage of MCF-7 and MDA-MB-231 cells 24 h post-treatment with test compounds (ABZ, PU and ABZ-PU) at different concentrations (5, 15, 25, 50 and 100 µM). Cell viability was normalized at corresponding concentrations of DMSO-treated cells. (Statistical significance: *: *p* < 0.05, **: *p* < 0.01, ***: *p* < 0.001 and ****: *p* < 0.0001).

As shown in Figure 10, the lowest concentration (5 µM) of ABZ induced a slight proliferative effect on the cell population of both types of tumor cell lines, as follows: a viability percentage of 107.66% on MCF7 cells and 108.5% on MDA-MB-231 cells. However, the concentration of 15 µM ABZ induced a lower proliferative activity (104.83%) only on the ER+ cell line (MCF7 cells), whereas the cell viability of ER− cell line (MDA-MB-231 cells) was decreased to 86.57%.

Nevertheless, the two cell lines exposed to higher concentrations (25, 50, 100 µM) of ABZ behaved quite differently; the viability of ER+ cells decreased in a dose-dependent manner, with a viable cell population of 85.17%, 78.85% and 65.93%, respectively, whereas the ER− cell line displays a plateau pattern with cell viability values of 84.34%, 82.56% and 81.57%, respectively. Regarding the PU sample, both cell lines presented good viability rates (above 90%) after exposure to all test concentrations. For the sample of interest (ABZ-PU), the lowest two concentrations (5 and 15 µM) induced very slight viability impairment, both cell lines manifesting above 90% viabilities. However, the ER+ cell line was more affected in terms of viability after treatment with concentrations of 25, 50 and 100 µM of ABZ-PU, respectively, compared to the ER− cell line treated with the same concentrations. The viability percentages of MCF7 cells after treatment with the highest three concentrations of ABZ-PU were as follows: 84.22%, 82.84% and 64.3%, respectively while the viability rates of MDA-MB-231 cells subjected to the same treatments are 89.08%, 78.96% and 73%, respectively. 

#### 2.8.2. Fluorescent Microscopy

**Figure 11:**
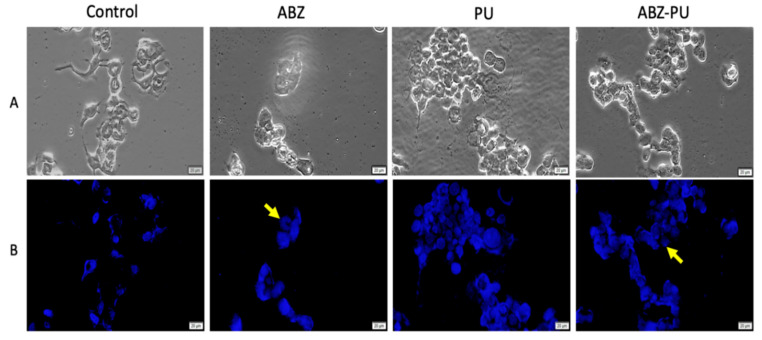
MCF7 cells stimulated with the highest concentration (100 µM) of test samples (ABZ, PU, ABZ-PU) for 24 h. (**A**)—Bright field microscopy was used to observe the cell morphology; (**B**)—Hoechst staining was employed to assess the specific apoptotic markers (chromatin condensation/DNA fragmentation). Scale bars represent 20 µm.

**Figure 12:**
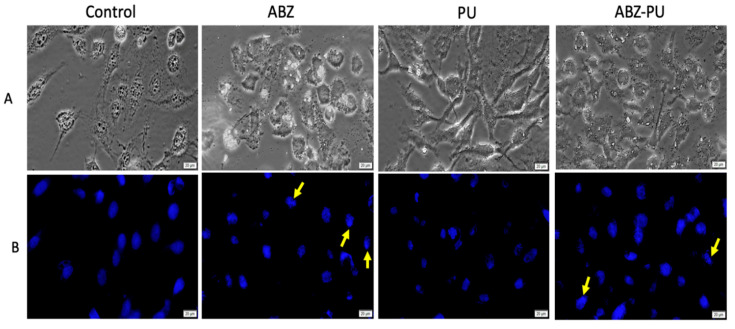
MDA-MB-231 cells stimulated with the highest concentration (100 µM) of test samples (ABZ, PU, ABZ-PU) for 24 h. (**A**)—Bright field microscopy was used to observe the cell morphology; (**B**)—Hoechst staining was employed to assess the specific apoptotic markers (chromatin condensation/DNA fragmentation). Scale bars represent 20 µm.

In order to further evaluate the cellular morphological changes and DNA alterations, both cell lines were exposed to the highest concentration (100 µM) of test samples that induced a significant cell viability decrease as noticed through the MTT assay. The cells were analyzed under bright field and fluorescent microscopy (Figure 11 and Figure 12).

As presented in Figure 11A, MCF7 cells exposed to 100 µM of ABZ and ABZ-PU, respectively, displayed cell confluence decrement and significant morphological alterations. Moreover, Figure 11B shows (marked with yellow arrows) that the cells treated with ABZ and ABZ-PU manifest several specific signs of apoptosis such as nuclei shape changes (ABZ-treated cells) and chromatin condensation (ABZ-PU-treated cells). However, the cells stimulated with empty PU did not express significant changes in terms of cell morphology and DNA alterations.

The most important morphological alterations of MDA-MB-231 cells were recorded after treatment with 100 µM ABZ for 24 h (Figure 12A). MDA-MB-231 cells undergoing specific signs of apoptosis were depicted in Figure 12B; the apoptotic markers were indicated with yellow arrows. One can notice that ABZ and ABZ-PU-treated cells manifest several apoptotic features, whereas PU-treated cells show no significant morphological alterations or apoptosis induction.

## 3. Discussion

Albendazole is a benzimidazole anthelminthic agent whose mechanism of action related to microtubule inhibition led to its reposition for extensive research as an antitumor agent [10]. The notable anti-proliferative activity of ABZ against several tumor cell lines was highlighted by numerous researches in the field [28]. ABZ has microtubule disrupting properties [29], inhibits angiogenesis by targeting the vascular endothelial growth factor (VEGF) expression [30] and is a potent inducer of apoptosis [4]. Furthermore, ABZ can inhibit glycolysis in cancer cells via phosphoenol pyruvate carboxykinase inhibition [31] and can determine the inhibition of hypoxia inducible factor (HIF-1α) [32]. Moreover, the drug has the ability to promote oxidative stress against tumor cells, by increasing ROS production and damaging the mitochondrial metabolic enzymes [33,34].

The major drawback in using ABZ in cancer therapy as a conventional pharmaceutical formulation consists in its erratic and low bioavailability, mainly due to its poor water solubility [8]. Many approaches have been conducted in order to improve the water solubility and consequently the bioavailability of albendazole, such as complexion with cyclodextrins, preparation of solid dispersions with polyvinylpyrrolidone, the use of surfactants [2,27], or the synthesis of ABZ-chitosan microparticles [35]. Also, anticancer ABZ-based formulations were obtained such as thiolated carboxymethyl chitosan-graft-β-cyclodextrin NP which showed good entrapment efficiency of ABZ and mucoadhesive properties [36], a nanosystem based on folate-conjugated bovine serum albumin with potent anti-tumor activity both in vitro and in vivo [32], ABZ-loaded albumin NP with antiproliferative properties on ovarian cancer cells [4], solid lipid NP with antitumor activity against glioma cell lines [15], inclusion complex with hydroxypropyl-beta-CD with cytotoxicity on ovarian cancer cells [5], and chitosan-tripolyphosphate NP with anticancer effect on cancer hepatic cells [16]. In addition, encapsulation in a nanocarrier may provide efficient and/or targeted delivery of the chemotherapeutic agent into the cancer cells [14,16] via endocytosis [37].

Polymeric NP are nowadays considered advanced drug delivery systems, offering various therapeutic advantages: multiple administration options, permeability of the target cells, enhanced therapeutic effect, protection of active principle, and feasible preparation methods [19,38,39,40]. Polymeric NP based on polyurethane gained a large interest in therapy due to their ability to meet many requirements for biomedical applications, such as biodegradability and easy elimination from the human body, thus facilitating repeated dosage and avoiding cumulative toxicity [41]. The benefits of PU nanocarriers were already investigated by researchers; Paiphansiri et al. [42] determined an increased uptake of PU microstructures in HeLa cells, Rosenbauer et al. [43], Padois et al. [44] and Danciu et al. [45] showed the potential of using PU as controlled- release carriers for anticancer and antimicrobial agents. Song Ni-Jia et al. have developed a PU targeting delivery system decorated with transtuzumab as a targeting ligand, with efficient cell entry and enhanced anticancer efficacy both in vitro and in vivo [46]. Morral-Ruiz et al. have successfully developed biotinylated-polyurethane-urea nanoparticles for the fluorescent detection of human hepatocellular carcinoma cells [47].

This study is aiming to provide valuable information for the design and antitumor in vitro investigation of a PU-based delivery system for ABZ, with improved ability to target cancer cells.

The method employed for the preparation of polymeric NP with specific properties influences their entrapment efficiency and ability to target cancer cells. Currently, the literature describes a multitude of preparation techniques classified as one-step and two-step procedures, mainly depending on the polymer particular parameters [48,49]. We synthesized the polyurethane particles by using an in situ polymerization technique, based on interfacial polycondensation combined with spontaneous emulsification previously described by Oprean et al. [50]. Stable PU nanoparticles, both unloaded and loaded with ABZ, were obtained with a mean size of 112 nm and 128 nm, respectively.

The newly obtained NP were evaluated using different analyses techniques, with recognized utility in the field of nanotechnology. The PU nanoparticles unloaded and loaded with ABZ were characterized in terms of size and homogeneity, stability, morphology (SEM) and encapsulation efficiency as well as in vitro biological effect.

The UV-Vis evaluation revealed a high encapsulation efficiency (62.4%), in agreement with previous studies which indicated polyurethane nanostructures as suitable carriers for active agents: 67.9% was found in the case of a chili pepper extract [51], and 82.9% for a ginger extract [52].

The recorded NP size is in direct correlation with their biological properties, influencing their circulation time, half-life, degradation, immunogenicity and cellular up-take. The literature highlighted that nanoparticles with sizes between 100 and 200 nm are capable of avoiding the reticulo-endothelial system (RES), in contrast to bigger NP that will be easily detected and eliminated [53,54]. Moreover, particles within this size range have a lower risk of immunogenicity and are not subjected to embolization when administered intravenously [49]. Furthermore, considering the numerous fenestrations described around a tumor, particles ranging between 100 and 200 nm were shown to have the advantage of accumulating in the tumor environment due to the enhanced permeability and retention effect (EPR) [47,55]. In terms of cellular up-take, a diameter larger than 100 nm allow particles to avoid the bone marrow caption [56]; therefore, we can state that ABZ-PU NP display the proper size to meet the requirements for a favorable pharmacokinetic profile and tumor accumulation.

The recorded values of the polydispersity index (0.2 for empty PU NPs and 0.3 for ABZ-PU NP) indicated that ABZ-PU NP are homogeneous and have a narrow size distribution, in complete agreement with the literature specifications [57].

The zeta potential is an important parameter that indicates the surface charge of particles from colloidal suspensions; it allows the prediction of particles’ stability against the tendency to form clusters or conglomerates. The literature states that stable particles are characterized by zeta potential values between −30 and +30 mV [58]. The zeta potential also provides useful data for particle assessment in terms of cellular uptake, biodistribution and interaction with other biological environments [59]. The PU NP were characterized by positive low values of the zeta potential, 32 mV for empty PU NP and 35 mV for ABZ-PU NP thus showing good stability and cell penetrability as well as a low capacity to activate the immune system [49]; positively charged particles have enhanced electrostatic interactions with the negatively charged cell membrane [60] which favor their internalization, as opposed to negatively charged or neutral particles which slow down the diffusion of the active drug into the cell matrix [54,61]. In addition, negatively charged NP are strongly retrieved by the RES system [61]. An increase of the zeta potential values of the drug-loaded nanoparticles compared to empty ones could be attributed to the adsorption of the drug to the NP’s surface [62]; however, in our experiment the measured values of the zeta potential for the two types of PU NP are very close to each other thus suggesting that ABZ adsorption to the NP surface through hydrogen bonds was negligible.

Thermal analysis is an important tool for the assessment of solid substances and commonly includes differential scanning calorimetry (DSC), thermogravimetry (TG) and derivative thermogravimetry (DTG); it provides an accurate insight into the solid state changes and energetic properties [63]. In particular, DSC analysis is the most comprehensive method to assess the formation of a new system, enabling observations that indicate possible interactions between individual components: shifts of the melting point, changes in the peak shape/area and fusion enthalpy [63].

The DSC analysis for the ABZ-PU NP showed the disappearance of ABZ melting point thus indicating the amorphization of pure drug, phenomenon able to improve its solubility and bioavailability, and its inclusion inside the PU envelope. Several minor differences recorded between the DSC profiles of loaded and unloaded PU NP could be attributed either to the existence of free drug adsorbed at the nanoparticles’ surface or to the direct influence of the encapsulated drug on the PU thermal profile.

In the TG/DTG analysis, one can notice a more rapid mass loss of the ABZ-PU NP as compared to pure ABZ, indicating an augmentation of the specific surface area of the former, compared to the latter. The results provided by the DSC, TG and DTG analysis confirms a reduction of the particle size and ABZ amorphization as a result of the encapsulation process. Hot-stage microscopy was used as a complementary technique to the DSC analysis; it enables the physical solid-state characterization of materials, as a function of temperature and time. The images allow an accurate explanation of the nature of the recorded thermal behavior [63]; by corroborating HSM micrographs with the corresponding DSC curves, one can confirm the formation of a new system in which ABZ is embedded in PU nanoparticles, with a small amount of pure ABZ adsorbed onto their surface.

Scanning electron microscopy is a surface imaging method often used to characterize nanoparticles; it provides valuable information regarding their size, size distribution and shape [64]. SEM images displayed ABZ-PU NP with approximately smooth surface, in accordance with PDI results, thus supporting the formation of a new solid phase in which ABZ is included inside PU nanoparticles. The SEM results confirmed that the newly synthesized NP are uniformly distributed and within the nano size range.

The FTIR analysis has been used in order to determine the solid state interaction between the pure drug and the nanocarrier; the method is commonly used to reveal insights upon the conjugation/encapsulation/adsorption of the active ingredient [64]. The comparative analysis of the FTIR spectra of pure materials and ABZ-PU nanoparticles revealed that few weak characteristic peaks of ABZ (1712.79, 1327.03, 1195.87 and 597.93 cm^−1^) were detected in the FTIR spectrum of ABZ-PU nanoparticles thus indicating the presence of non-encapsulated ABZ. However, the absence of high-intensity bands for albendazole in the ABZ-PU spectrum suggests that most of the ABZ was incorporated into the core of PU nanoparticles. Also, no new absorption bands were detected in the spectrum of ABZ-PU NP thus suggesting that ABZ did not affect the PU structure and stability; these results are in agreement with other previous reports [55,65].

In accordance with the above, the PXRD analysis also showed that traces of non-incorporated ABZ are present in the ABZ-PU formulation, as revealed by the recorded spectra. In addition, the morphological analysis of the final formulation does not indicate any noticeable presence of free ABZ in the ABZ-PU nanoparticle population; collectivelly, the physicochemical analytical results (PXRD, FTIR, DSC and SEM) indicate that the free ABZ is present in the final formulation only in trace amounts which could be attached to the nanoparticle surface, most likely through hydrogen bonding.

The PU matrices were formulated in order to encapsulate the ABZ drug whose poor water solubility was incriminated for its low bioavailability. In vitro dissolution studies performed in acidic (simulated gastric medium) and neutral (simulated intestinal medium) environments, respectively, indicated a better dissolution profile of pure ABZ in simulated gastric medium compared to the intestinal one; our results are consistent with previously published data which indicated a dramatic solubility decrease for ABZ as the pH of the environment increases [66] thus emphasizing the strong dependency between pH values and ABZ aqueous solubility.

ABZ-PU nanoparticles were also tested in order to assess the potential sustained release profile of the encapsulated drug; polymer nanoparticles may enhance the bioavailability of a drug in several situations among which poor dissolution profile and/or permeability issues [67]. Given its dissolution profile, after oral administration albendazole should prolong its residence within the gastric medium which is able to ensure a higher solubilization of ABZ compared to the intestinal medium with neutral pH values where the dissolution of the drug dramatically decreases. Therefore, a sustained release of ABZ within the acidic environment at stomach level would improve the overall absorption profile of the drug, subsequently improving its bioavailability for systemic therapeutic use. An improved solubilization profile occurred for the loaded ABZ versus pure drug when tested in neutral medium while in gastric medium the cumulative drug release was lower for the ABZ-PU nanoparticles compared to the pure drug. The ABZ-PU nanoparticles were able to provide a sustained release over a 24 h period in both acidic and neutral media; however, a solubility behavior matching the limits reported in the literature occurred only in the simulated gastric medium: 10–30% drug release in the first 2 h, 50% drug release within 8 h and 80% at the end of the 24 h interval [68]. For the simulated intestinal medium, the cumulated drug release was less than the stipulated percentages for all the mentioned time intervals presumably due to the poor solubility of ABZ at higher pH values. Marslin et al. reported in 2017 the preparation of ABZ-loaded solid lipid nanoparticles (SLN) which induced the slow release of the drug over a 24 h time period in phosphate buffer (pH = 7.4) reaching an 82% cumulative drug release [15]. Another study was published by Liu et al. in 2013 regarding the synthesis of ABZ-loaded chitosan nanoparticles which were tested in acidic, slightly acid and neutral environments, respectively; the chitosan-nanoparticles revealed a similar behavior to our nanoformulations with the drug’s highest dissolution in acid medium and a sustained release of the drug following encapsulation [69]. Similar results were also reported by Panwar et al. in 2010 who prepared ABZ-loaded liposomes which revealed the sustained release of the drug over a 4.5 h period in slightly acid phosphate buffer (pH = 5.6) [70]. Therefore, one could state that our in vitro dissolution results are consistent with the previously reported experimental data regarding other types of nanoformulations; also, the ABZ-PU nanoparticles are suitable for the design of a controlled drug release oral administration. In addition, the dissolution studies provided us with some information regarding the strength of the bonds formed between the drug and the encapsulation PU matrix; thus, the matrix-drug interactions are strong enough to allow the nanoparticles’ isolation following synthesis but also weak enough to ensure ABZ release in simulated biological environment. Moreover, the spectra drawn for each collected sample over the entire UV-Vis range of wavelengths revealed the same absorption maximum value as the one recorded for pure ABZ, thus indicating the chemical stability of the drug following the encapsulation process as well as its lack of impurities. Therefore, from a pharmaceutical point of view, we may consider the formulated nanoparticles as potential future therapeutic options.

In recent years, numerous in vitro and in vivo studies have provided results that support the applicability of the broad-spectrum anti-helminthic ABZ against various types of cancer [71,72,73,74,75].

In the present paper, the cytotoxic effect of a new ABZ-PU formulation was tested in estrogen receptor positive (ER+) human breast adenocarcinoma MCF-7 cell line and estrogen receptor negative (ER−) MDA-MB-231 cell line. A previous study reported by Jaydam et al. assessed the in vitro antiproliferative activity of ABZ on MCF-7 breast cancer cell line [76]. In agreement with the findings of this study, the results of our work showed a significant dose-dependent decrease of cell viability upon treatment with ABZ (Figure 8). Intriguingly, this dose-dependent effect was significant only when test samples were applied on the MCF-7 breast cancer cell line. In addition, the cell viability inhibition in MDA-MB-231 cell lines followed a plateau pattern despite the administration of increasing doses, reaching significant inhibitory effects only when the highest ABZ concentration was applied (100 µM). Taken together, one can assume that the potent anti-proliferative effect displayed by ABZ has a preferential activity against the ER+ breast cancer cell line.

In order to overcome the low bioavailability of ABZ, recent scientific efforts are focused on finding new formulations able to increase its effectiveness as a systemic anticancer agent. Positive results were reported by the group of Noorani et al. who developed an albumin-based nanoparticle formulation cross-linked with glutaraldehyde [4]. The newly synthesized ABZ nanoparticles (200–300 nm) proved to have a high drug loading capacity and a sustained drug release profile. The use of nanosystems as an approach to increase ABZ bioavailability was also employed by the group of Liang [32] who revealed that ABZ-loaded albumin silver nanoparticles effectively inhibited the proliferation of MCF-7 cell lines in a dose-dependent manner. Moreover, the ABZ nanosystem suppressed tumor growth without any obvious organ toxicity in the MCF-7 tumor-xenografted nude mouse model [32]. The newly PU-based nanoparticle delivery system for ABZ developed in our study produced a significant decrease of cell viability when tested at the highest concentration (100 µM, Figure 8) in both MCF-7 (35.7%) and MDA-MB-231 cell lines (27%) vs. control (DMSO treated cells). In addition, ABZ-PU 100 µM impaired cellular viability in both ER+ and ER− tumor cell lines in a higher degree than exerted by ABZ alone: 34.07% in ER+ and 18.43% in ER− cell lines (Figure 8). As expected, PU alone, in all tested concentrations, did not decrease cellular viability (Figure 8) thus providing biocompatible properties. These results suggest that the new ABZ-PU formulation increases the drug’s potential as an anticancer agent, in particular against the ER− breast cancer cell line. Similar results were obtained with a well-known anticancer drug with limited hydrosolubility, paclitaxel; the study pursued the synthesis of a waterborne PU micellar nanoformulation and evaluation of its effects and delivery capacity in MCF-7 breast cancer cells [77]. Results revealed that PU alone had no cytotoxic effect in treated cells, whereas paclitaxel alone exhibited a mild cytotoxic effect, significantly lower in comparison to that of the paclitaxel—loaded PU nanomicelles [77].

The most successful non-invasive treatment against all types of cancers is induction of apoptosis and cell death. Any drug that can activate through various mechanisms the apoptotic pathway or inhibit the endogenous anti-apoptotic molecules is a strong candidate to become an efficient anticancer agent. As reported by a great number of studies, ABZ can induce apoptosis through various mechanisms; in human gastric cancer cells, the ABZ anticancer activity emerged due to its ability to interfere with microtubule formation and function, causing mitotic arrest and subsequently inducing apoptosis [78]. The same microtubule disrupting ability was employed as anticancer treatment by Patel et al. in small cell lung cancer and metastatic melanoma cell lines [8]. Another recent work reported that ABZ modulates ROS (reactive oxygen species) production, causes oxidative damage to DNA and finally triggers apoptosis and induces cell death in human breast cancer cells, as well as in Ehrlich ascitic carcinoma cells [10]. In our current study, the morphological hallmarks of apoptosis were assessed by means of Hoechst 33342 staining in MCF-7 (Figure 9) and MDA-MB-231 (Figure 10) cell lines, upon treatment with PU, ABZ and the novel ABZ-PU formulation (100 µM). The recorded data showed that MCF7 cells exposed to ABZ and ABZ-PU underwent significant morphological alterations, such as nuclei shape changes (ABZ-treated cells) and chromatin condensation (ABZ-PU-treated cells), events accompanied by pyknosis and pseudopod retraction. Moreover, there were no significant cell morphology changes and DNA alterations in either MCF-7 or MDA-MB-231 cells stimulated with PU nanoparticles alone. In regards to the MDA-MB-231 cell line, upon stimulation with both ABZ and ABZ-PU, one could notice the same apoptosis-related morphological changes. These results indicate that in terms of the antitumor underlying mechanisms, ABZ and ABZ-PU are able to induce apoptosis in breast cancer cells; the ABZ-PU formulation not only did not hinder but increase ABZ anticancer potency, enabling its more effective use as antitumor therapy in breast cancer. This effect is particularly important in triple negative breast cancers such as the MDA-MB-231 tumor cell line that proved to be highly refractory to conventional treatment or to rapidly develop drug resistance. Future studies are planned in order to reveal the intimate molecular mechanisms behind the anticancer activity of ABZ as well as the influence of PU encapsulation on the cellular uptake of the drug.

## 4. Materials and Methods

### 4.1. Chemicals and Reagents

ABZ analytical standard (purity > 98%) was a gift sample obtained from Biesterfeld GmbH (Hamburg, Germany) and was used without further purification. Isophorone diisocyanate (IPDI) and acetone were purchased from Merck (Hohenbrunn, Germany); polycaprolactone (PCL, average Mn ~14,000) and Span^®^85 were purchased from Sigma Aldrich (Steinheim, Germany). Monoethylene glycol (EG) was purchased from Lach-Ner s.r.o. (Neratovice, Czech R.) and 1,4-butanediol (BD) was purchased from Carl Roth GmbH (Karlsruhe, Germany). All substances were used as received.

### 4.2. Preparation of Polyurethane Microstructures

Polyurethane particles were synthesized using a multi-step procedure based on interfacial polycondensation technique combined with spontaneous emulsification, as previously described in the literature [18,50].

Briefly, the organic phase (2.0 mL IPDI mixed with 30.0 mL acetone, heated at 35 °C and homogenized at 350 rpm for at least 10 min) was injected into the aqueous phase (2.0 mL EG, 2.0 mL BD, 1.0 mL PCL and 1.0 mL Span^®^85 mixed with 40.0 mL distilled water, heated at 35 °C and homogenized at 350 rpm for at least 10 min). The injection of organic phase into the aqueous phase was made under magnetic stirring 550 rpm and heated at 55 °C for 4 h in order to ensure the complete formation of polymeric walls.

The products were repeatedly washed with a mixture of water/acetone (1:1 *v/v*) in order to remove by-products or traces of unreacted precursors and then were maintained in Petri dishes in thin layers for approximately 10 days, for water and acetone removal. The resulting powder was stored in glass vials, at room temperature, for further investigation.

Albendazole was separately introduced (in a ratio of 5–15%) in the organic phase, in one experiment, while the second one, representing empty polymeric particles, was used as control.

### 4.3. Encapsulation Efficiency

The binding affinity or the adherence efficacy of a drug delivery system is probably the most important parameter in this field; it has been established by reporting the quantity of free ABZ to the total amount that was used during the synthesis, following a procedure previously reported (Equation (1)) [79]:

(1) $$\mathrm{BA}=\frac{\mathrm{TA}-\mathrm{FA}}{\mathrm{TA}}\times 100$$

where: BA is the binding affinity, TA is the total amount of ABZ and FA represents the quantity of free ABZ that was identified in the last step of synthesis (the purification by repeated wash-centrifugation cycles).

The UV-Vis spectra and the maximum absorption values were recorded on an UVi Line 9400 Spectrophotometer (SI Analytics, Mainz, Germany). An important difference was noticed between the maximum absorption of ABZ (235 nm) and that of carrier structures (305 nm), allowing the accurate identification of the two substances. A calibration curve was plotted between 0.05 and 5.00 µg/mL ABZ and used to determine the values of TA and FA, respectively, by using the Beer-Lambert law.

### 4.4. Evaluation of Particle Size and Polydispersity Index

For the evaluation of particle size and polydispersity index a Vasco Size Analyzer (Cordouan Tech., Pessac, France) was used, with the following operating conditions: temperature 25 °C; time interval 70–95 μs; number of channels 580–670; laser power 75–90%; DTC position—UP; acquisition mode—continuous, and analysis mode—Pade-Laplace. The size distribution of the obtained polyurethane particles was measured using ethanol solutions of 1:100 (*w/w*). The measurements were done in triplicate and an average value was considered.

### 4.5. Zeta Potential

The surface charge of polyurethane particles was measured using a Wallis Zeta potential Analyzer (Cordouan Tech., Pessac, France). The following parameters were selected: cuvette type-plastic; temperature 25 °C; multiple measurements-minimum 5; solvent-water; resolution-medium, 0.8 Hz and Henry function (Smoluchowski).

### 4.6. Thermal Analysis

#### 4.6.1. DSC/TG/DTG

The thermal behavior of the mentioned samples was monitored using a Netzsch STA 449 C instrument (Netzsch Holding, Selb, Germany). A mass between 16–17.5 mg sample was placed in an alumina crucible and then the temperature was raised from 25 to 1000 °C with 10 °C/min, under artificial air flow of 20 mL/min.

#### 4.6.2. Hot-Stage Microscopy

Microscopic observations of thermal behavior of materials and their changes during heating were carried out with a MZ 6 Thermomicroscope (Leica, Bensheim, Germany). The samples were observed under the microscope by using a scanning speed of 2 °C/min. The magnification in the photographs was 59.7×.

### 4.7. Morphological Characterization

Particle morphology was assessed by scanning electron microscopy (SEM) (Hitachi S4700, Hitachi Scientific Ltd., Tokyo, Japan) at 10 kV. The samples were gold-palladium coated (90 s) with a sputter coater (Bio-Rad SC 502, VG Microtech, Uckfield, UK) using an electric potential of 2.0 kV at 10 mA for 10 min. The air pressure was 1.3–13.0 mPa.

### 4.8. Spectroscopic Characterization

#### 4.8.1. FTIR Spectroscopy

The FTIR study was carried out using a Prestige-21 spectrometer (Shimadzu, Duisburg, Germany). The FTIR spectra of pure ABZ, PU and ABZ-PU particles, respectively, were measured using the potassium bromide pellets method. Small amounts of samples were grinded with potassium bromide and the mixture was pressed into a disk. The FTIR spectra were recorded over the region 4000–400 cm^−1^, at a resolution of 4 cm^−1^.

#### 4.8.2. X-ray Spectroscopy

Crystal structure of the excipients and the microparticles were analyzed by a Bruker D8 Advance diffractometer (Bruker AXS GmbH, Karlsruhe, Germany). The experiments were performed in symmetrical reflection mode with Cu Kα1 radiation (λ = 1.5406 Å), using Göbel Mirror bent gradient multilayer optics. The scattered intensities were measured with a Våntec-1 line detector. The angular range was from 3° to 40° in steps of 0.007°. Other measurement conditions were as follows: target, Cu; filter, Ni; voltage, 40 kV; current, 40 mA; measuring time, 0.1 s per step.

### 4.9. In Vitro Disolution Studies

In vitro dissolution studies were conducted by means of a modified Erweka DT device (Erweka, Langen, Germany), at 37 ± 2 °C, in 100 mL simulated gastric/intestinal medium, at 100 rpm. 5 mL samples were collected in each case at 15′ and 30′ and then every hour for a 24 h interval in order to evaluate the release profile of ABZ. The ABZ concentration of each sample was assessed spectrophotometrically by means of ATI Unicam UV/VIS spectrometer at 292 nm for the simulated gastric medium and 296 nm for the simulated intestinal medium where ABZ reached the absorption maximum. Calibration curves were constructed for each dissolution medium and allowed the calculation of the solubilized ABZ. All tests were conducted in triplicate in order to gain statistical value.

The simulated gastric/intestinal media were freshly prepared according to the following compositions:

Simulated gastric medium: sodium chloride (NaCl) 0.35 g, chlorhydric acid (HCl) 1N 94.0 g, glycocol 0.50 g, distilled water up to 1000 mL (pH = 1.10).

Simulated intestinal medium: anhydrous disodium phosphate 14.40 g, monopotassium phosphate 7.10 g, distilled water up to 1000 mL (pH = 7.00).

In order to evaluate the chemical stability of ABZ as a result of the encapsulation procedure, spectra were drawn for each collected sample over the entire UV-Vis range of wavelengths (200–400 nm).

### 4.10. Anti-Proliferative and Apoptosis Assay

#### 4.10.1. Cell Culture

Two genotypically different human breast adenocarcinoma cell lines were used as follows: MCF7 cells (ATCC^®^ HTB-22TM) were used as the estrogen receptor positive (ER+) cell line and MDA-MB-231 cells (ATCC^®^ HTB-26TM) were used as the estrogen receptor negative (ER−) cell line. Both types of cell lines were acquired from American Type Culture Collection (ATCC, Manassas, VA, United States). The specific cell culture media used for MCF7 cells was Eagle’s Minimum Essential Medium (EMEM) supplemented with 15% Fetal Calf Serum (FCS), while MDA-MB-231 cells were cultured in high glucose Dulbecco’s Modified Eagle’s Medium (DMEM) enriched with 10% FCS. Both cell culture media were supplemented with an antibiotic mixture of 0.1 mg mL^−1^ streptomycin/100 IU mL^−1^ penicillin to avoid a possible fungal/microbial contamination of the cell cultures. The specific culture media (EMEM and DMEM) were supplied by ATCC while FCS and antibiotic mixture were provided by Sigma-Aldrich (Munich, Germany). MTT assay kit was acquired from Roche Diagnostics GmbH (Mannheim, Germany), while Hoechst staining was purchased from Thermo Fisher Scientific (Waltham, MA, USA).

#### 4.10.2. MTT Assay

As a primary screening, the colorimetric 3-4,5-dimethylthiazol-2-yl)-2,5-diphenyltetrazolium bromide (MTT) assay was employed to evaluate the viability percentage of both human breast adenocarcinoma cell lines (MCF-7 and MDA-MB-231) after exposure to five different concentrations (5, 15, 25, 50, 100 µM) of test samples (ABZ, PU, ABZ-PU) after an incubation period of 24 h. Dimethyl sulfoxide (DMSO) was used as a solvent to obtain the stock solutions of the test samples. In brief, the protocol consisted in seeding 1 × 10^4^ cells/well in 96-well plates and allowing them to attach and to reach the optimal confluence. Afterward, the old medium was removed through suction using an aspiration station and the cells were treated with 100 µL/well of fresh medium containing the test samples at a final concentration of 5, 15, 25, 50, 100 µM and further incubated for a period of 24 h. The control cells were exposed to the same amount of DMSO, which was used to obtain the five test concentrations from the stock solutions. After the stimulation period has ended, 10 µL/well MTT reagent was added for a period of 3 h, protected from light. In this period the viable cells, through mitochondrial reductase, converted MTT to dark blue formazan crystals and the precipitated crystals were further solubilized with 100 µL/well of lysis buffer solution. In order to quantify the cell viability, the absorbance of each well was spectrophotometrically determined at a wavelength of 570 nm using a microplate reader (xMark^TM^ Microplate Spectrophotometer, Bio-Rad Laboratories, Inc., Hercules, CA, USA).

#### 4.10.3. Hoechst Staining

The cells (MDA-MB-231 and MCF7) were cultured on cover slips in 6-well plates at an initial cell density of 5 × 10^6^ cells/well. When the optimal confluence was reached, the cells were stimulated with test samples at concentration of 100 µM and they were further incubated for a period of 24 h. Afterwards, the cells were washed with 1.5 mL PBS/well, fixed with 4% paraformaldehyde in PBS, washed again with PBS and treated with 500 µL/well of Hoechst working solution. After this step, the plates were incubated at room temperature for approximately 10 min, in a dark chamber. At the end, the cells were washed with 1.5 mL/well PBS to remove the excess of staining solution. Thereafter, the cover slips were mounted on glass slides using Fluoromont^TM^ medium (Sigma-Aldrich, Munich, Germany) and the cells were analyzed under an IX73 inverted microscope (Olympus, Tokyo, Japan) equipped with ultraviolet filter.

### 4.11. Statistical Analyses of Data

For the in vitro studies, the results were expressed as mean ± standard deviation (SD) of three independent experiments. The data were subjected to statistical analysis by one-way analysis of variance (ANOVA) followed by Tukey’s posttest. Differences between sample treated cells and DMSO treated cells were considered significant when *p* < 0.05, *p* < 0.01, *p* < 0.001, *p* < 0.0001 indicated by *, **, ***, **** respectively.

## 5. Conclusions

In the present study we have obtained ABZ-encapsulated polyurethane nanoparticles through the interfacial polycondensation technique which modulated in a positive manner particle parameters such as shape and size as well as the morphology of the pure drug. The physicochemical analysis revealed significant morphological changes in the shape and aspect of ABZ-PU nanoparticles with respect to the pure components, thus supporting the formation of a new solid phase occurred due to the encapsulation of ABZ in the PU particles. In addition, the interactions between ABZ and PU in solid state were assessed through spectroscopic and thermal techniques.

The biological tests conducted on the pure drug and its PU nanoformulations, respectively, revealed a significant antiproliferative activity on the ER+/− breast cancer cell lines exhibited by pure ABZ which increased as a result of entrapment into the PU nanoparticles. Deeper investigations indicated apoptosis induction followed by cell death as the underlying mechanisms of the antitumor activity recorded for both ABZ and its PU nanoformulations. The lack of toxicity reported for the pure PU material stands as evidence for its potential as a biocompatible nanocarrier for anticancer drugs.

However, the current study has its limitations, the most important being the lack of in vivo biological data. Future studies are needed in order to confirm the potential clinical use of ABZ and its nanoformulations in cancer treatment; nonetheless, the in vitro data reported in the current paper provide a starting point for the development of a new therapeutic option in the therapy of breast cancer.
